# Endovascular Treatment for Acute Stroke Patients With a Pre-stroke Disability: An International Survey

**DOI:** 10.3389/fneur.2021.714594

**Published:** 2021-10-04

**Authors:** Sanjana Salwi, Jan A. Niec, Ameer E. Hassan, Christopher J. Lindsell, Pooja Khatri, J. Mocco, Jeffrey L. Saver, Eva A. Mistry

**Affiliations:** ^1^School of Medicine, Vanderbilt University, Nashville, TN, United States; ^2^Department of Neurology, University of Texas Rio Grande Valley, Harlingen, TX, United States; ^3^Department of Biostatistics, Vanderbilt University Medical Center, Nashville, TN, United States; ^4^Department of Neurology and Rehabilitation Medicine, University of Cincinnati, Cincinnati, OH, United States; ^5^Department of Neurosurgery, Mount Sinai Health System, New York, NY, United States; ^6^Department of Neurology, University of Calfornia, Los Angeles, Los Angeles, CA, United States

**Keywords:** ischemic stroke, endovascular treatment, acute stroke, treatment, survey, disability

## Abstract

**Background:** It is unclear what factors clinicians consider when deciding about endovascular thrombectomy (EVT) in acute ischemic stroke patients with a pre-existing disability. We aimed to explore international practice patterns and preferences for EVT in patients with a pre-stroke disability, defined as a modified Rankin score (mRS) ≥ 2.

**Methods:** Electronic survey link was sent to principal investigators of five major EVT trials and members of a professional interventional neurology society.

**Results:** Of the 81 survey-responding clinicians, 57% were neuro-interventionalists and 33% were non-interventional stroke clinicians. Overall, 64.2% would never or almost never consider EVT for a patient with pre-stroke mRS of 4-5, and 49.3% would always or almost always offer EVT for a patient with pre-stroke mRS 2-3. Perceived benefit of EVT (89%) and severity of baseline disability (83.5%) were identified as the most important clinician-level and patient-level factors that influence EVT decisions in these patients.

**Conclusion:** In this survey of 80 respondents, we found that EVT practice for patients with pre-stroke disability across the world is heterogenous and depends upon patient characteristics. Individual clinician opinions substantially alter EVT decisions in pre-stroke disabled patients.

## Introduction

The American Heart Association/American Stroke Association national practice guidelines for management of acute ischemic stroke only recommend offering endovascular thrombectomy (EVT) to patients with an acute ischemic stroke involving a large anterior cerebral vessel occlusion without a pre-stroke disability, defined as pre-stroke modified Rankin Score (mRS) of 0 or 1 (1). However, about a third of acute ischemic stroke patients are disabled at pre-stroke baseline (2, 3). Small, observational studies have shown that: (1) almost one in three patients undergoing EVT in routine clinical practice has a baseline pre-stroke disability, and (2) when treated with EVT, patients with pre-stroke disability have similar odds of retaining their baseline function as do patients without pre-stroke disability (4–6). To better understand current EVT care approaches for pre-stroke disabled patients without guideline recommendations, it is important to study individual clinician and institutional level practice and preferences regarding EVT in patients with pre-stroke disability. We conducted a survey of international experts in EVT and practitioners of interventional neurology to understand how EVT decisions are made in routine clinical practice for patients with pre-stroke disability. Here, we describe factors influencing physician decisions when considering EVT for patients with pre-existing disability, including the impact of level and type of disability, additional patient characteristics, and institutional protocols informing EVT treatment decisions.

## Materials and Methods

### Survey Design

A 20-item survey was developed by the authors based on factors most frequently cited in prior literature as influential in the EVT decision-making process. A pilot version was completed by 24 physicians at two institutions. Feedback from respondents was used to improve survey readability and accuracy. The final survey questionnaire in its entirety is provided in the Supplementary Material. The survey had inbuilt logic where participants could proceed with answering follow-up questions relevant to and dependent on their answers to a prior question. Participants were first asked if they would ever consider EVT in patients with pre-stroke mRS 2 and pre-stroke mRS 3. If they answered no, their responses for pre-stroke mRS 4-5 were automatically also handled as no. If they answered yes to either, they were asked to further rate the degree of their likelihood, on a 6-level Likert scale, of offering EVT to patients with pre-stroke mRS of 2-3 and patients with pre-stroke mRS of 4-5. Similarly, specifics of institutional patient selection protocols were only asked if a participant responded yes to having an institutional protocol on EVT practice. In addition, respondents were asked to identify among 14 general, patient-specific, and stroke-specific features those that were most important to their decision-making regarding offering EVT to individual patients with pre-stroke disability. As both benefits or harms of EVT are yet unknown for patients with a pre-stroke disability, we refer to each as “perceived” by the treating clinician.

### Survey Distribution

Invitations to complete the final survey were emailed to all author-investigators of the first five international pivotal EVT trials of stent retrievers and to members of the Society of Vascular and Interventional Neurology (SVIN) (7–11). For the authors of the international trials, two follow-up emails were sent if initial emails did not elicit a response. For SVIN members, no follow-up emails were sent as per Society policy. Study data were collected from January 8, 2020 to January 9, 2020 and managed using REDCap (Research Electronic Data Capture) electronic data capture tools hosted at Vanderbilt University Medical Center (12, 13). Survey responses were de-identified and analyzed across three major domains- current perspectives on how pre-stroke disability level and type, patient characteristics, and institutional factors impact decision to treat.

### Analysis

Survey responses were de-identified. Survey respondent characteristics were characterized using frequencies (numerator/denominator) for each response option. Ordinal three-level frequency distributions of physician likelihood of pursuing EVT [(1) always/almost always, (2) often/sometimes, (3) almost never, never of pursuing EVT] were calculated separately for the two scenarios of patients with pre-stroke disability of mRS 2-3 and patients with pre-stroke disability of 4-5 and separately for non-interventionalists and interventionalists. Frequencies of physician selection of multiple-choices among 14 response options for general, patient-specific, and stroke-specific features most important to EVT decision-making in patients with pre-stroke disability were calculated and reported in rank order from most to least frequent.

## Results

### Respondent Characteristics

Completed survey responses were received from a total of 81 physician-experts, including 33 from among the international EVT trial authors and 48 from among the SVIN society members. The response rate among the international EVT trial authors total was 33/119 (28%). The denominator for the SVIN survey recipients was unavailable due to use of email listserv technical constraints.

The characteristics of survey physician respondents is shown in Table 1. Among the 81 respondents, the majority identified as male (71/81, 87.7%). The most common specialties were interventional vascular neurology and non-interventional vascular neurology, followed by interventional neuroradiologists, with smaller proportions of neurohospitalists, vascular neurosurgeons, general neurologists, and emergency medicine physicians. The majority of respondents had practice experience of more than 10 years duration, and a little over a quarter between 5 and 10 years, with one-sixth having <5 years. The most common academic ranks were Full Professor and Associate Professor, followed by Assistant Professor, with Fellows accounting for just over 1 in 10. Overall, proportion of clinical time dedicated to stroke care was over half in 71.6% and between 10 and 50% in another 25.9%. No two respondents were from the same institution. There were no missing data for this analysis.

**Table 1:** Demographics of survey respondents.

***n*** **= 81 (%)**	
Male	71 (87.7)
**Years in practice**	
<5 years	12 (14.8)
>10 years	47 (58.0)
5-10 years	22 (27.2)
**Primary specialty**	
Emergency medicine	1 (1.2)
General neurology	1 (1.2)
Interventional neuro-radiology	10 (12.3)
Interventional vascular neurology	36 (44.4)
Neuro hospitalist	3 (3.7)
Neurocritical care	1 (1.2)
Non-interventional vascular neurology	27 (33.3)
Other	1 (1.2)
Vascular neurosurgery	1 (1.2)
**Academic rank**	
Assistant Professor	15 (18.5)
Associate Professor	22 (27.2)
Fellow	10 (12.3)
Instructor	1 (1.2)
Other	10 (12.3)
Professor	23 (28.4)
**Proportion of time dedicated to caring for stroke patients**	
0-10%	2 (2.5)
11-50%	21 (25.9)
51-99%	46 (56.8)
100%	12 (14.8)

### Perspectives on Pre-stroke Disability and Treatment

The frequency with which physicians indicated they would ever consider EVT for an otherwise eligible patient was: 97.5% for pre-stroke mRS 2; 79% for mRS 3; and 74% for mRS 4-5.

**Figure 1:**
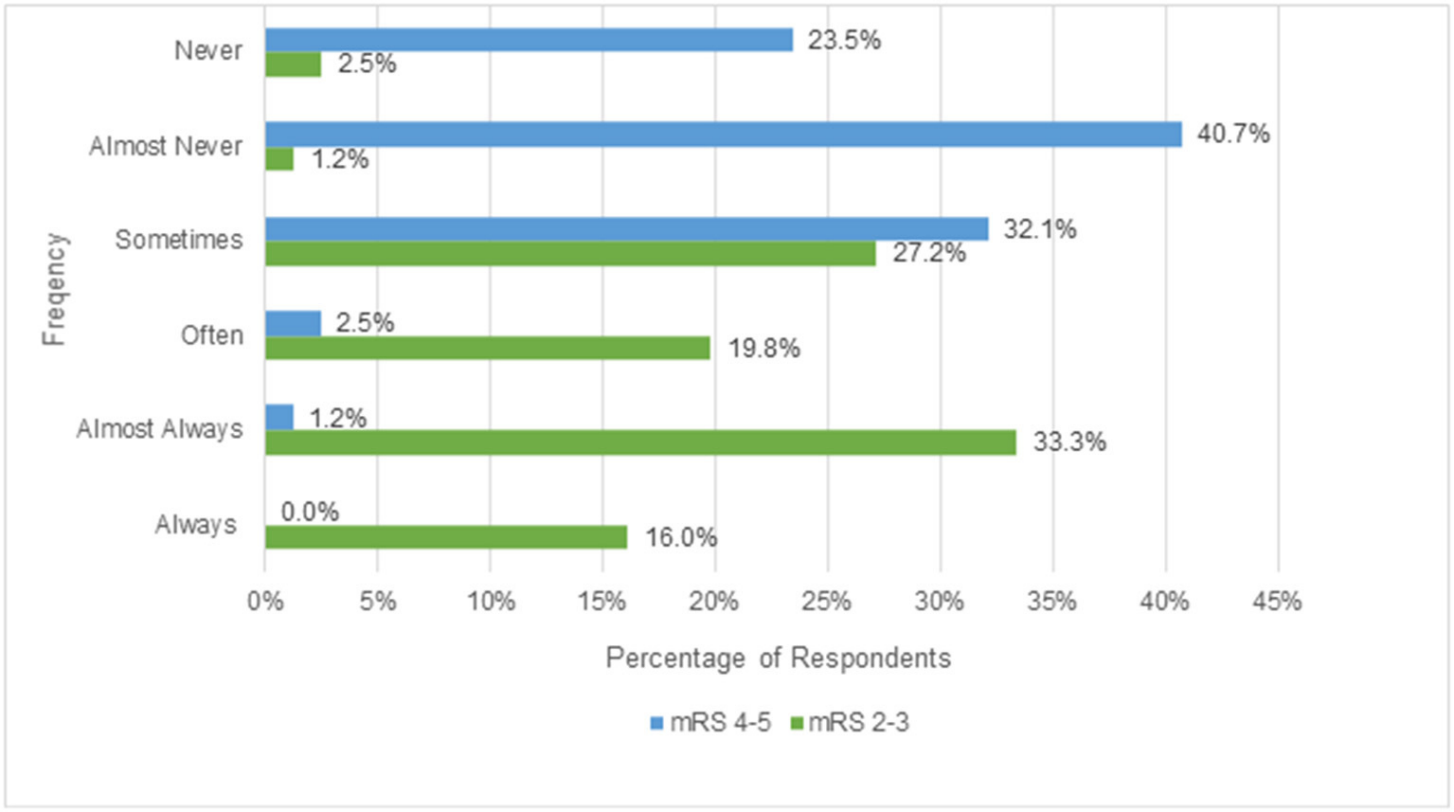
Physician likelihood of considering EVT for patients with pre-stroke disability of mRS 2-3 vs. 4-5.

The degrees of physician likelihood of considering, performing, or offering EVT for patients with pre-stroke disability of mRS 2-3 and patients with pre-stroke disability of 4-5 are shown in Figure 1. For patients with mRS 2-3, a total of 49.3% (40/81) would always or almost always offer EVT; 47.0% (38/81) would often or sometimes offer EVT; and 3.8% (3/81) would almost never or never offer EVT. In contrast, for patients with pre-stroke mRS of 4 or 5, a total of 1.2% (1/81) would always or almost always offer EVT; 34.6% (28/81) would often or sometimes offer EVT; and 64.2% (52/81) would almost never or never offer EVT. Table 2 shows the degree of physician likelihood of offering EVT separately for neurointerventionalists and non-interventionalists. Overall, neurointerventionalists were mildly more likely than non-interventionalists to offer EVT to both patients with pre-stroke disability of mRS 2-3 and patients with pre-stroke disability of mRS 4-5. Supplementary Table 1 provides a detailed breakdown by subspecialty of the likelihood with which respondents indicated they would consider EVT for mRS 2-3 and EVT for mRS 4-5.

**Table 2:** Frequency of EVT for mRS 2-3 and mRS 4-5 by subspecialty.

**Frequency of EVT**	**Neuro-interventionalists** **(***n*** = 47)**	**Non-interventionalists** **(***n*** = 34)**
**Pre-stroke mRS 2-3**		
Always	19%	12%
Almost always	32%	36%
Often	23%	15%
Sometimes	26%	29%
Almost never	0%	0%
Never	0%	3%
**Pre-stroke mRS 4-5**		
Always	0%	0%
Almost always	0%	3%
Often	0%	0%
Sometimes	38%	24%
Almost never	43%	39%
Never	19%	32%

Most respondents considered the complication risk (60/79, 76%) and the likelihood of recanalization (73/79, 92%) to be the same between patients with vs. without pre-stroke disability. However, most physicians thought patients with pre-existing disability had a lower rate of return to baseline disability (53/79, 67%).

**Figure 2:**
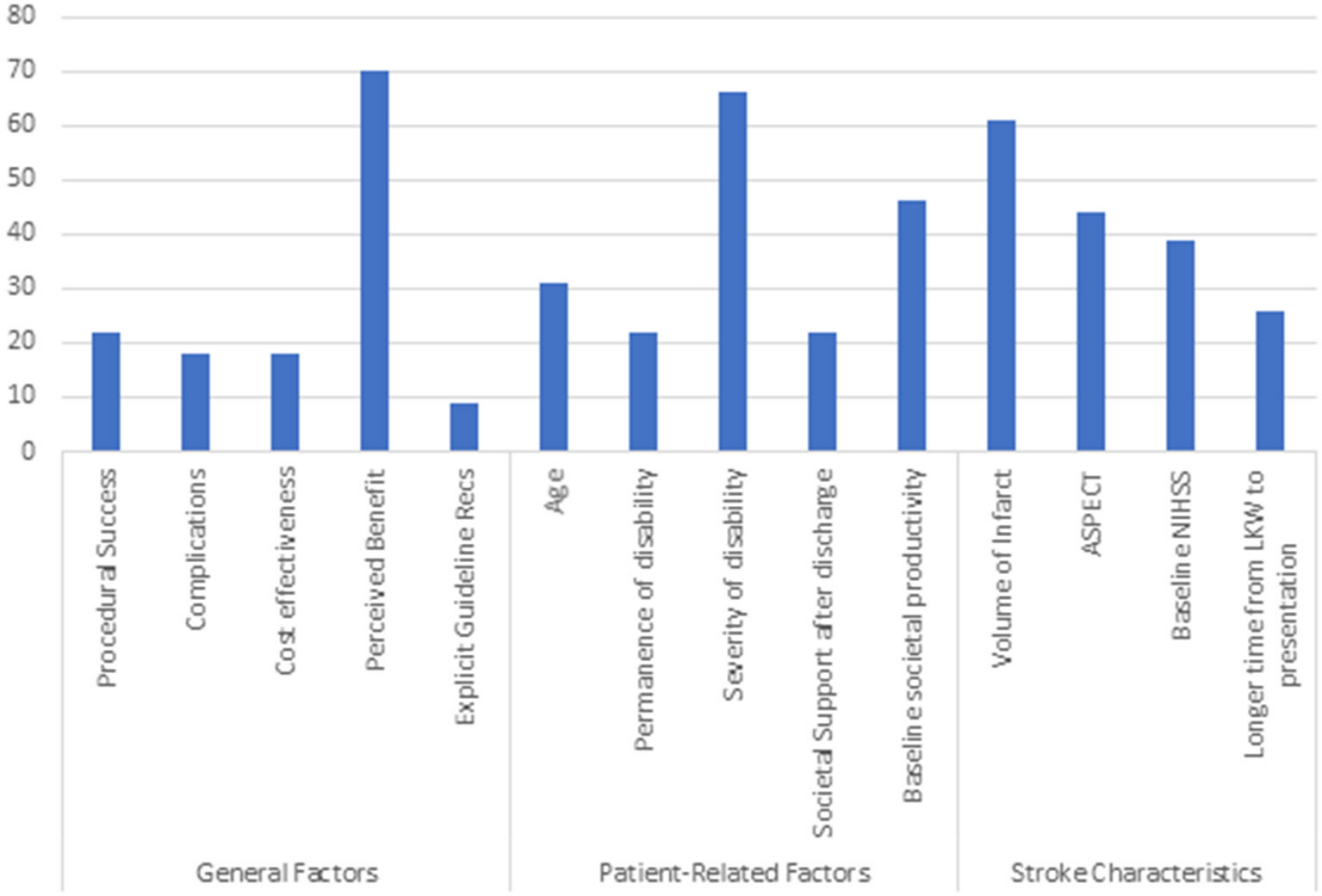
Factors affecting EVT decision-making.

Respondents were asked to identify among 14 general, patient-specific, and stroke-specific features those that were most important to their decision-making regarding offering EVT to individual patients with pre-stroke disability. Among the 79 respondents who indicated they would ever offer EVT to a patient with pre-stroke mRS 2-5, Figure 2 shows the frequency with which they identified 14 general, patient-specific, and stroke specific features as most important to their decision-making regarding offering EVT to individual patients with pre-stroke disability. Perceived benefit was the factor that physicians took into consideration most often when deciding whether to offer an EVT to a patient with pre-existing disability (70/79, 89%). Among patient-related factors that influenced decision making, 66 physicians chose severity of disability (83.5%), 46 chose baseline societal productivity (58.2%), and 31 chose age (39%) as important factors. Only 22 (28%) physicians chose either societal support and 22 (28%) permanence of disability as important factors. Among stroke-specific characteristics, volume of infarct was the factor that physicians most often cited as influential (61/79, 77%).

### Institutional Practices

A substantial majority of physicians (65/81, 80%) responded that their institution had general guidance on how to select patients for EVT. Of those reporting institutional guidance, most indicated that protocols did not take a stand on how to treat patients with pre-existing disability and left the decision up to individual physicians (36/65, 55%). Only five institutions had explicit guidance on how to treat patients with pre-existing disability with three recommending to never treat and two recommending to always treat patients with pre-existing disability. The remaining 24 organizations providing any direction advised that patients with pre-stroke disability were sometimes candidates for EVT.

## Discussion

We observed considerable individual clinician and institutional-level heterogeneity in EVT practice among acute ischemic stroke patients with a pre-existing disability in this survey of 80 respondents. Disposition to pursue treatment was strongly influenced by the level of patients' pre-existing disability. While nearly half of the survey respondents noted that they would always or almost always treat patients with mild-to-moderate pre-existing disability, a preponderance of respondents noted they would almost never or never do so for patients with severe disability. In addition to the wide variation in overall propensity to treat, physicians varied substantially when identifying which general, patient-specific, and stroke-specific characteristics were important to them when making treatment decisions in individual patients with pre-stroke disability.

The current AHA/ASA guidelines only recommend offering EVT to acute ischemic stroke patients who do not have a pre-existing disability (1). This guideline is based on the completed randomized trials assessing EVT efficacy, which excluded patients with a pre-stroke mRS 2-5 (7–11). Patients who are disabled at pre-stroke baseline are typically excluded from initial pivotal acute stroke clinical trials because the trials measure success of acute stroke therapies by the extent to which they reduce all-cause disability after stroke. Pre-stroke disabled patients are less informative for this outcome as their pre-stroke disability constrains the degree to which the acute stroke treatment can affect final all-cause disability. However, because a sizeable proportion of acute ischemic stroke patients are disabled prior to their index stroke, a detailed understanding of pre-stroke disability sources, pre-stroke disability functional profiles, ideal outcome measures, and presence and magnitude of treatment effects of acute stroke treatments such as EVT are urgently needed to ensure inclusive design of future acute stroke research and rapid translation of therapeutic advances into care of disabled patients. A detailed understanding of the current landscape of the EVT practice in this population could significantly aid such future, large, multicenter, prospective studies.

The present study not only highlights heterogeneity in EVT practice among patients with pre-stroke disability, but offers some insights into factors influencing individual clinician reasoning in this practice. While most of the survey respondents believed that the complication risks and success of recanalization were the same for patients with pre-stroke disability as those without pre-stroke disability, the majority also believed that patients with pre-stroke disability would be less likely to return to their baseline disability. Almost 90% of physicians considered their own valuation, or perception, of benefit to patient as a major factor in deciding whether to offer EVT to patients with disability. It is important to note that, in the absence of comparative data on benefits and risks of EVT compared with medical management, clinician perception is based on physiologic reasoning (as noted by majority of the physicians choosing baseline volume of infarct and Alberta Stroke Program Early CT, ASPECT, score as the most important stroke characteristic for offering EVT) and personal experience, rather than definitive randomized clinical trial evidence. Accordingly, the findings of this study reflect the current range of opinion and intuition among individual clinicians making EVT decisions for pre-stroke disabled patients and underscore the need for more reliable, formal evidence.

Importantly, large variation in institutional guidance for EVT in patients with pre-existing disability is highlighted in our study. The majority of institutional protocols remain silent on EVT specifically pertaining to pre-stroke disable patients and leave clinical decision making up to individual clinicians. Of the 6% of institutions with strict guidance on offering EVT to patients with existing disability, half recommended to always offer and half recommended to never offer EVT to pre-stroke disabled patients, highlighting inter-institutional heterogeneity in practice.

Our study has several limitations. First, respondents of our survey were drawn from lead investigators in international EVT trials and members of a US professional society largely populated by interventional neurologists. Therefore, our results may not be generalizable to the entire population of physicians that perform endovascular interventions for acute ischemic stroke. Second, the response rate to survey invitations was moderate and it is possible that the views of non-respondents would have differed from those of respondents. The strengths of our study include responses from physicians in nine different subspecialties with a good mix of practice experience, academic rank, and geographic diversity.

## Conclusions

In this International survey of 80 respondents, we found considerable individual clinician and institutional-level heterogeneity in EVT practice among acute ischemic stroke patients with a pre-existing disability. Likelihood to offer EVT differed according to baseline disability as well as patient and stroke characteristics. Further research into effects of EVT among patients with a pre-stroke disability is warranted. Such research should utilize novel study methodologies and outcome measures to overcome challenges of studying this patient population.

## Data Availability Statement

The raw data supporting the results of this study will be made available upon a reasonable request.

## Ethics Statement

Ethical review and approval was not required for the study on human participants in accordance with the local legislation and institutional requirements. Written informed consent from the participants was not required to participate in this study in accordance with the national legislation and the institutional requirements.

## Author Contributions

SS contributed to data collection, analysis, and drafting of the manuscript. JN contributed to data collection. AH contributed to data collection and critical revision of the manuscript. CL, PK, JM, and JS contributed to conceptualization and critical revision of the manuscript. EM contributed to data collection, drafting of manuscript, and conceptualization. All authors contributed to the article and approved the submitted version.

## Funding

EM received salary support from NIH/NINDS (K23NS113858) for this work.

## Conflict of Interest

The authors declare that the research was conducted in the absence of any commercial or financial relationships that could be construed as a potential conflict of interest.

## Publisher's Note

All claims expressed in this article are solely those of the authors and do not necessarily represent those of their affiliated organizations, or those of the publisher, the editors and the reviewers. Any product that may be evaluated in this article, or claim that may be made by its manufacturer, is not guaranteed or endorsed by the publisher.

## References

[1] Powers WJ Rabinstein AA Ackerson T Adeoye OM Bambakidis NC Becker K. Guidelines for the early management of patients with acute ischemic stroke: 2019 update to the 2018 guidelines for the early management of acute ischemic stroke: a guideline for healthcare professionals from the American Heart Association/American Stroke Association. Stroke. (2019) 50:e344–418. 10.1161/STR.0000000000000211 31662037

[2] Karlinski M Kobayashi A Czlonkowska A Mikulik R Vaclavik D Brozman M. Safe Implementation of Treatments in Stroke-Eastern Europe (SITS-EAST) Investigators. Role of preexisting disability in patients treated with intravenous thrombolysis for ischemic stroke. Stroke. (2014) 45:770–5. 10.1161/STROKEAHA.113.003744 24496395

[3] Ganesh A Luengo-Fernandez R Pendlebury ST Rothwell PM. Long-term consequences of worsened poststroke status in patients with premorbid disability. Stroke. (2018) 49:2430–6. 10.1161/STROKEAHA.118.022416 30355105 PMC6159688

[4] Goldhoorn R-JB Verhagen M Dippel DWJ van der Lugt A Lingsma HF Roos YBWEM. Safety and outcome of endovascular treatment in prestroke-dependent patients. Stroke. (2018) 49:2406–14. 10.1161/STROKEAHA.118.022352 30355090

[5] Regenhardt RW Young MJ Etherton MR Das AS Stapleton CJ Patel AB. Toward a more inclusive paradigm: thrombectomy for stroke patients with pre-existing disabilities. J NeuroInterv Surg. (2020) 13:865–8. 10.1136/neurintsurg-2020-016783 33127734 PMC8365380

[6] Salwi S Cutting S Salgado AD Espaillat K Fusco MR Froehler MT. Mechanical thrombectomy in patients with ischemic stroke with prestroke disability. Stroke. (2020) 51:1539–45. 10.1161/STROKEAHA.119.028246 32268851 PMC7367056

[7] Berkhemer OA Fransen PSS Beumer D van den Berg LA Lingsma HF Yoo AJ. A randomized trial of intraarterial treatment for acute ischemic stroke. N Engl J Med. (2015) 372:11–20. 10.1056/NEJMoa1411587 25517348

[8] Jovin TG Chamorro A Cobo E de Miquel MA Molina CA Rovira A. Thrombectomy within 8 hours after symptom onset in ischemic stroke. N Engl J Med. (2015) 372:2296–306. 10.1056/NEJMoa1503780 25882510

[9] Saver JL Goyal M Bonafe A Diener H-C Levy EI Pereira VM. Stent-retriever thrombectomy after intravenous t-PA vs. t-PA alone in stroke. N Engl J Med. (2015) 372:2285–95. 10.1056/NEJMoa1415061 25882376

[10] Nogueira RG Jadhav AP Haussen DC Bonafe A Budzik RF Bhuva P. Thrombectomy 6 to 24 hours after stroke with a mismatch between deficit and infarct. N Engl J Med. (2017) 378:11–21. 10.1056/NEJMoa1706442 29129157

[11] Albers GW Marks MP Kemp S Christensen S Tsai JP Ortega-Gutierrez S. Thrombectomy for stroke at 6 to 16 hours with selection by perfusion imaging. N Engl J Med. (2018) 378:708–18. 10.1056/NEJMoa1713973 29364767 PMC6590673

[12] Harris PA Taylor R Thielke R Payne J Gonzalez N Conde JG. Research electronic data capture (REDCap)—a metadata-driven methodology and workflow process for providing translational research informatics support. J Biomed Inform. (2009) 42:377–81. 10.1016/j.jbi.2008.08.010 18929686 PMC2700030

[13] Harris PA Taylor R Minor BL Elliott V Fernandez M O'Neal L. The REDCap consortium: Building an international community of software platform partners. J Biomed Inform. (2019) 95:103208. 10.1016/j.jbi.2019.103208 31078660 PMC7254481

