# Supplementary material for: Endovascular Treatment for Acute Stroke Patients With a Pre-stroke Disability: An International Survey
Source: Front Neurol. 2021 Oct 4;12:714594. doi: 10.3389/fneur.2021.714594 (PMC8520928; doi:10.3389/fneur.2021.714594)
Supplement: Supplementary file 1 [file Table_1.DOCX]

Table S1. Frequency of EVT for mRS 2-3 and mRS 4-5 by Subspecialty

| Frequency of EVT | Interv Neurol  (n=36) | Non-Interv  Vasc Neurol  (n=27) | Interv Neurorad  (n=10) | Neurohospitalist  (n=3) | Vasc Nsurg  (n=1) | Gen Neurol  (n=1) | Neurocritical Care (n=1) | Emergency Medicine (n=1_ |
| --- | --- | --- | --- | --- | --- | --- | --- | --- |
| Pre-stroke mRS 2-3 | | | | | | | |  |
| Always | 22% | 11% | 10% | 0% | 0 | 100% | 0 | 0 |
| Almost Always | 36% | 37% | 20% | 67% | 0 | 0 | 0 | 0 |
| Often | 19% | 19% | 40% | 0% | 0 | 0 | 0 | 0 |
| Sometimes | 22% | 26% | 30% | 33% | 100% | 0 | 100% | 0 |
| Almost Never | 0% | 4% | 0% | 0% | 0 | 0 | 0 | 0 |
| Never | 0% | 4% | 0% | 0% | 0 | 0 | 0 | 100% |
| Pre-stroke mRS 4-5 | | | | | | | |  |
| Always | 0% | 0% | 0% | 0% | 0 | 0 | 0 | 0 |
| Almost Always | 0% | 4% | 0% | 0% | 0 | 0 | 0 | 0 |
| Often | 0% | 0% | 0% | 0% | 0 | 0 | 0 | 0 |
| Sometimes | 42% | 22% | 30% | 0% | 0 | 100% | 0 | 0 |
| Almost Never | 42% | 41% | 50% | 33% | 0 | 0 | 100% | 0 |
| Never | 17% | 33% | 20% | 67% | 100% | 0 | 0 | 100% |
