# Supplementary material for: Endovascular Treatment for Acute Stroke Patients With a Pre-stroke Disability: An International Survey
Source: Front Neurol. 2021 Oct 4;12:714594. doi: 10.3389/fneur.2021.714594 (PMC8520928; doi:10.3389/fneur.2021.714594)
Supplement: Supplementary file 2 [file Data_Sheet_1.PDF]

Pre-stroke Disability and Mechanical Thrombectomy in Acute Stroke Patients

Please complete the following survey. Thank you!

What is the name of your institution?	______________________
What is your full name (first_last)?	______________________
What is your gender?	◯ Female<br>◯ Male<br>◯ Other
How many years have you been practicing as a stroke specialist?	◯ < 5 years<br>◯ 5-10 years<br>◯ >10 years
What is your primary practice speciality?	◯ Non-interventional Vascular Neurology<br>◯ Interventional Vascular Neurology<br>◯ Vascular Neurosurgery<br>◯ Interventional Neuro-radiology<br>◯ General Neurology<br>◯ Neuro Hospitalist<br>◯ Emergency Medicine<br>◯ Neurocritical Care<br>◯ Other
What is your current academic rank?	◯ Fellow<br>◯ Instructor<br>◯ Assistant Professor<br>◯ Associate Professor<br>◯ Professor<br>◯ Other
What proportion of your current clinical practice is dedicated to caring for stroke patients?	◯ 0-10%<br>◯ 11-50%<br>◯ 51-99%<br>◯ 100%
Would you typically consider/perform/offer mechanical thrombectomy (MT) to otherwise eligible acute stroke patients with a pre-existing disability (modified Rankin Score, mRS, of ≥2)?	◯ Yes<br>◯ No/Never<br>(Modified Rankin Score is graded as: 0=no symptoms, 1=minimal but functionally independent and able to carry out all activities, 2=functionally independent but unable to carry out all activities, 3=functionally dependent but able to walk without assistance from another individual, 4= functionally dependent and requires significant assistance to ambulate, and 5=bedridden)

Would you typically consider/perform/offer mechanical thrombectomy (MT) to otherwise eligible acute stroke patients with a pre-stroke modified Rankin Score, mRS, of ≥3?	◯ Yes<br>◯ No/Never<br>(Modified Rankin Score is graded as: 0=no symptoms, 1=minimal but functionally independent and able to carry out all activities, 2=functionally independent but unable to carry out all activities, 3=functionally dependent but able to walk without assistance from another individual, 4= functionally dependent and requires significant assistance to ambulate, and 5=bedridden)
How often would you consider/perform/offer offer MT to otherwise eligible acute stroke patients with a pre-stroke mRS of 2 to 3?	◯ Always<br>◯ Almost always<br>◯ Often<br>◯ Sometimes<br>◯ Almost Never<br>◯ Never
How often would you consider/perform/offer offer MT to otherwise eligible acute stroke patients with a pre-stroke mRS of 4 to 5?	◯ Always<br>◯ Almost always<br>◯ Often<br>◯ Sometimes<br>◯ Almost Never<br>◯ Never
What do you judge is the risk of complications (such as symptomatic hemorrhage) with MT in disabled patients, when compared to those WITHOUT pre-existing disability?	◯ Same risk in patients with or without pre-existing disability (if otherwise similar patient and imaging characteristics)<br>◯ Risk is higher in patients with pre-existing disability<br>◯ Risk is lower in patients with pre-existing disability
What do you judge is the rate of successful recanalization with MT in disabled patients, when compared to those WITHOUT pre-existing disability?	◯ Same rate in patients with or without pre-existing disability<br>◯ Rate is higher in patients with pre-existing disability<br>◯ Rate is lower in patients with pre-existing disability
What do you judge is the rate of return to the baseline function with MT in disabled patients, when compared to those WITHOUT pre-existing disability?	◯ Same rate in patients with or without pre-existing disability<br>◯ Rate is higher in patients with pre-existing disability<br>◯ Rate is lower in patients with pre-existing disability
What are the most important factors that you take into consideration when making a decision to consider/perform/offer MT to patients with pre-existing disability? (Check all that apply)	☐ Procedural success<br>☐ Complications<br>☐ Cost effectiveness<br>☐ Perceived benefit<br>☐ Explicit guideline recommendations
Which are the most important individual patient characteristics that influences your decision to consider/perform/offer MT in patients with pre-existing disability? (Check all that apply)	☐ Age<br>☐ Permanence of disability<br>☐ Severity of disability (moderate vs severe)<br>☐ Social support after discharge<br>☐ Baseline societal productivity and/or quality of life despite disability

Which are the most important concurrent stroke characteristics that you take into consideration when making a decision to consider/perform/offer MT to patients with pre-existing disability? (Check all that apply)	☐ Volume of infarct<br>☐ Baseline CT brain ASPECT score<br>☐ Baseline NIH stroke scale score<br>☐ Longer time from last known well to the presentation<br>☐ Other (Please specify)
Other (please specify)	__________________________
Does your institution have an acute stroke treatment protocol with GENERAL guidance on patient selection for MT?	◯ Yes<br>◯ No
Does this protocol recommend offering/performing MT for otherwise eligible acute stroke patients who have a pre-existing disability?	◯ It recommends to ALWAYS treat patients with pre-existing disability<br>◯ It recommends to SOMETIMES treat patients with pre-existing disability<br>◯ It recommends NEVER to treat patients with pre-existing disability<br>◯ It does not take a stand on this aspect of patient selection and allows for individual considerations
Additional comments/considerations pertaining to MT in stroke patients with pre-existing disability	__________________________________
